# A Study of Self-Care Practice in Routine Radiotherapy Care: Identifying Differences Between Practitioners and Non-Practitioners in Sociodemographic, Clinical, Functional, and Quality-of-Life-Related Characteristics

**DOI:** 10.1177/15347354221130301

**Published:** 2022-10-15

**Authors:** Kerstin Stake-Nilsson, Silje Gustafsson, Kristina Tödt, Per Fransson, Anna Efverman

**Affiliations:** 1 University of Gävle, Gävle, Sweden; 2 Luleå University of Technology, Luleå, Sweden; 3 Skåne University Hospital, Sweden; 4 Umeå University, Umeå, Sweden

**Keywords:** cancer care, category scale, complementary and alternative medicine, effect moderators, Numeric Rating scale, nursing, oncology care, rehabilitation, treatment expectations, Visual Analog scale

## Abstract

**Objectives:**

The objective of this study was to describe self-care practice during radiotherapy for cancer and to identify potential differences between practitioners and non-practitioners of self-care regarding sociodemographic, clinical, functional, and quality-of-life-related characteristics.

**Methods:**

In this descriptive study, 439 patients (87% response rate) undergoing radiotherapy responded to a study questionnaire regarding self-care, sociodemographic, clinical (eg, experienced symptoms), functional, and quality-of-life-related characteristics.

**Results:**

Of the 439 patients, 189 (43%) practiced at least one self-care strategy, while 250 (57%) did not. In total, the patients described 332 self-care practices, resulting in 14 different categories of self-care strategies. The 5 most common indicators of practicing self-care were fatigue, general wellbeing, psychological symptoms, nausea, vomiting and improving physical condition. The 5 most common self-care strategies were physical activity, increased recovery, healthy eating, distraction, and skincare. Patients who were married, were older than 69, patients with less education than university education, patients undergoing a combination of internal and external radiotherapy, patients experiencing fewer than 8 symptoms, and better quality of life, practiced self-care to a lower extent than did other patients. Functional capacity did not differ between self-care practitioners and non-practitioners.

**Conclusion and Implications for Practice:**

Of the patients undergoing radiotherapy, slightly less than half practiced self-care during an ordinary week of radiotherapy. Because older and less-educated patients were less likely to practice self-care, cancer care practitioners should consider paying particular attention to helping such patients with their self-care practice.

## Introduction

People undergoing cancer therapy face many challenges in everyday life that decrease their Quality of Life (QoL), for example, the burden of cancer-therapy-induced side effects.^1-11^ Each individual has his/her own way of dealing with these challenges, often by using non-pharmacological or self-care management strategies.^7^ Effective pharmacology medical therapies to reduce some of the side effects exist but are often costly^12^ and may produce drug interactions and additional side effects.^13^ Further, it has been found that a considerable proportion of patients avoid pharmacological therapies and desire non-pharmacological integrative therapies^7,14^ or self-care.^15^ Accordingly, integrative oncology is a patient-centered, evidence-informed field of cancer care that utilizes mind and body practices, natural products, or lifestyle modifications from different traditions, alongside conventional medical therapies.^16^

Regarding the everyday life challenges experienced by people undergoing cancer therapy, all cancer therapies can cause burdensome side effects, and patients experience several symptoms, which often occur in clusters and negatively affect their QoL.^8,9^ QoL often varies depending on health status and symptom experience; some researchers thus define aspects of QoL that are related to health as “health-related QoL.”^17^ Compared to the frequently studied chemotherapy-induced side effects,^1^ fewer studies have looked at the patient-reported side effects commonly experienced during radiotherapy.^4,6^ Healthcare practitioners often underestimate symptom occurrence and symptom burden during radiotherapy.^18^ The side effects experienced during radiotherapy depend on the type of radiotherapy and the target location of radiotherapy on the body. Fatigue, sleeping problems, nausea, and vomiting are commonly experienced and may be persistent if not adequately managed^4,10^ In addition, emotional side effects such as stress, depression, and anxiety have been frequently reported.^11^ Presence of pain, fatigue and anxiety had the strongest association with worsened QoL during radiotherapy.^9^ To decrease the negative consequences, management and reduction of cancer-therapy-induced side effects are increasingly important,^1,19^ and can be achieved using for example, self-care strategies.^20^

Self-care is a central concept in health care and may be regarded as the means through which a patient can maintain, restore, and improve health and well-being. The practice of self-care can also be expanded as a concept and fundamental goal for individuals to reach a high extent of self-care agency.^21^ Self-care strategies in cancer are highly divergent and individual, and they extend across multiple domains such as medicine and pharmacology, lifestyle, psychology, social support, knowledge and information, navigation, and coordination, and medical decision-making.^4,15,22^ Common integrative cancer therapies, among the wide range of adopted strategies, include dietary or nutritional and lifestyle modifications, physical activity, and increased recovery and relaxation.^20,23,27^ Self-care may include strategies used to help in coping with the side effects of cancer therapies, and the choice of such strategies is based on the individual patient’s preferences.^15,28,29^ Consequently, self-care as a health-promoting intervention may be important to consider as a potential method of reducing the severity of side effects. Effective use of self-care^20,24-27^ may relieve psychological distress and physical symptoms as well as offer cancer patients a feeling of control over their illness and symptoms. This could, in turn, enable them to play an active role in their healthcare, thus improving QoL and reducing suffering.^28^

Regarding the utilization of self-care, many different self-care strategies have been described,^15,22,26-29^ and considerable beneficial effects of self-care have been demonstrated in patients undergoing cancer therapy.^20,23-27^ However, the fact that there is scientific evidence for such effects based on randomized controlled trials does not mean that patients will adhere to practicing beneficial self-care strategies in the context of routine care. In many efficacy studies of self-care practice, patients have been guided by healthcare practitioners and e-health devices that enable them to practice adequate self-care.^24,26^ Implementation research has clearly shown that dissemination of new knowledge often does not suffice; to achieve implementation in routine practice that is marked by quality, a systematic approach to implementation is recommended, starting with an investigation of the target population’s perspectives, for example, the patient group of interest.^30^ Self-care practice for cancer-therapy-induced side effects in routine cancer care settings seems to have been studied only rarely. There would appear to be a lack of knowledge regarding the perspective of patients; Are they able, without guidance from health practitioners or e-health devices manage to practice self-care for symptoms commonly experienced during routine care radiotherapy? Do patients who practice self-care differ from non-practitioners? Adding knowledge that helps answer these questions would be useful in identifying and supporting subgroups of patients regarding self-care practice in integrative cancer care.

## The Aim

The aim was to study practice of self-care and to identify potential differences between practitioners and non-practitioners of self-care regarding sociodemographic, clinical, functional, and quality-of-life-related characteristics.

## Methods

### Design and Setting

The present exploratory, descriptive, cross-sectional questionnaire study covered patients at 4 oncology clinics, which have a total of 6 radiotherapy departments, located at Swedish university hospitals and regional hospitals in the southern, western, and eastern regions of Sweden. The study adhered to the Swedish research ethics law (2003:460) and the Declaration of Helsinki’s ethical principles for medical research involving human subjects. The regional ethical committee approved the study (reg.no. 2015/101-30).

### Inclusion

A study coordinator randomly selected one single day for data collection, that is, the “study day,” at each radiotherapy department. Preceding the study day, radiotherapy nurses at each radiotherapy division screened patients using the study criteria. The inclusion criteria were: patients 18 years or older, receiving fractionated external radiotherapy for cancer irrespective of diagnosis, and willingness to give informed consent. The exclusion criteria were: receiving the very first or single radiotherapy fraction on the study day, or having a physical, mental, or linguistic limitation of such severity that it hindered informed consent or study participation. Informed consent was obtained from all participants.

## Data Collection

### The Procedure, Development, and Testing of the Study Questionnaire

The patients completed a study questionnaire (Swedish) in private (ie, mostly at home/ward unit/patient hotel) using a digital web-based data form or paper and pen (self-preferred choice). The questionnaire included the clinimetric^31^ and psychometric measurements described below. Preceding the study, the clinimetric measures were developed based on patient interviews. The measures were satisfactorily tested for face validity (n = 20 patients undergoing radiotherapy, unpublished data) presenting that the measures were reasonable, easy to understand, and measured the phenomenon supposed to be measured, according to the target population. The clinimetric measures were then tested for test-retest reliability (n = 36 patients receiving radiotherapy, chemotherapy or therapies combined), observing that the Spearman’s correlation coefficients between test and re-test ranged .421 (walking by foot) to .984 (purchase food or other necessities) for the variety of included measures.^3^ Thereafter, the clinimetric measures were subsequently used in a previous study (n = 200 patients undergoing radiotherapy).^15^ Prior to the current study, we pilot-tested the study questionnaire, including the clinimetric and psychometric measures presented below, regarding feasibility (n = 55 patients undergoing radiotherapy, unpublished data). The pilot test demonstrated high response rates, supporting the questionnaire’s feasibility for use in the target population.

### Documenting Patients’ Sociodemographic and Clinical Descriptive Characteristics

Coordinating radiotherapy nurses collected clinical descriptive data from the patients’ medical records, for example, cancer diagnosis and accumulated dose of radiotherapy. In a study questionnaire, the patients detailed their sociodemographic and clinical background characteristics regarding cancer therapy and co-morbidities. The variables are presented in Table 1.

**Table 1.** Sociodemographic and Clinical Characteristics of the Study Participants, and Differences Between Practitioners.

Variable	Total, n = 439	Did not practice self-care n = 250	Practiced self-care n = 189	*P*-value univariable analyses (crude models)	Relative risk, 95% CI	*P* value multivariable analyses (adjusted model)
*Sociodemographic variables*						
Sex, n (%)	n = 439					
Female	223 (51)	110 (49)	113 (51)	Ref.	Ref.	Ref.
Male	216 (49)	140 (65)	76 (35)	<.001*	1.31, 1.11-1.55	.73
Age, n (%)	n = 435	n = 246	n = 189			
24-46	44 (10)	12 (27)	32 (73)	Ref.	Ref.	Ref.
47-68	198 (45)	96 (48)	102 (52)	.01*	1.78, 1.07-2.94	.09
69-90	193 (45)	138 (72)	55 (28)	<.001*	2.62, 1.60-4.28	<.001*
Marital status, n (%)	n = 439					
Living apart	21 (5)	8 (38)	13 (62)	Ref.	Ref.	Ref.
Married	330 (75)	193 (58)	137 (42)	.07	1.53, 0.88-2.67	.01
Living alone (single)	88 (20)	49 (56)	39 (44)	.15	1.46, 0.82-2.60	.34
Occupational status,^d^ n (%)	n = 437	n = 249	n = 188			NA^d^
Employed	42 (10)	21 (50)	21 (50)	.37	1.31, 0.70-2.45	
Sick leave	120 (27)	51 (42)	69 (58)	.70	1.12, 0.62-2.00	
Retired	254 (58)	169 (66)	85 (34)	.01*	1.75, 1.00-3.03	
Other^a^	21 (5)	8 (38)	13 (62)	Ref.	Ref.	
Educational level, n (%)	n = 430	n = 247	n = 183			
College/University	151 (35)	66 (44)	85 (56)	Ref.	Ref.	Ref.
Professional training	110 (25)	64 (58)	46 (42)	.02*	1.33, 1.05-1.69	.53
Secondary school	97 (23)	62 (64)	35 (36)	<.001*	1.46, 1.16-1.85	<.000*
Elementary school	72 (17)	55 (76)	17 (24)	<.001*	1.75, 1.00-3.03	<.001*
Native country, n (%)	n = 435	n = 248	n = 187			NA^b^
Sweden	385 (88)	219 (57)	166 (43)	Ref.	Ref.	
Other	50 (12)	29 (58)	21 (42)	.89	1.02, 0.79-1.31	
*Clinical characteristics*						
Cancer type, n (%)	n = 411	n = 233	n = 178			
Breast cancer	163 (40)	78 (48)	85 (52)	Ref.	Ref.	Ref.
Prostate cancer	142 (35)	96 (68)	46 (32)	<.001*	1.41, 1.16-1.72	.94
Head, neck, or brain cancer	54 (13)	30 (56)	24 (44)	.33	1.16, 0.87-1.55	.57
Gynecologic or colorectal cancer	42 (10)	20 (48)	22 (52)	1.00	0.99, 0.70-1.42	.11
Lymphoma	10 (2)	9 (90)	1 (10)	<.001**	1.88, 1.45-2.44	NA^c^
Tumor surgery preceding the external radiotherapy	n = 432	n = 167	n = 265			
Yes	265 (61)	139 (52)	126 (48)		Ref.	Ref.
No	167 (39)	107 (65)	60 (35)	.02	0.82, 0.69-0.96	.25
Types of cancer therapies, combined	n = 439	n = 250	n = 189			
External radiotherapy, only	231 (53)	138 (60)	93 (40)	.05	1.20, 0.99-1.45	.73
External and internal radiotherapy	45 (10)	31 (69)	14 (31)	.02	1.39, 1.07-1.78	.10
External radiotherapy and concomitant chemotherapy	149 (34)	74 (50)	75 (50)		Ref.	Ref.
External and internal radiotherapy, and concomitant chemotherapy	13 (3)	7 (54)	6 (46)	.77	1.08, 0.63-1.83	.69
Cancer metastasis, n (%)	n = 401	n = 228	n = 173			NA^b^
Yes	59 (15)	32 (54)	27 (46)		Ref.	
No	342 (85)	196 (57)	146 (43)	.66	1.06, 0.82-1.36	
Co-morbidity, any other diseases than cancer, n (%)	n = 410	n = 231	n = 179			NA^b^
No	227 (55)	128 (56)	99 (44)	Ref.	Ref.	
Yes	183 (45)	103 (56)	80 (44)	1.00	0.10, 0.84-1.19	
Number of symptoms, n (%)	n = 395	n = 225	n = 170			
≤7	291 (74)	177 (61)	114 (39)	.01*	1.32, 1.05-1.65	.12
≥8	(26)	48 (46)	56 (54)		Ref.	Ref.
Accumulated radiation period	n = 430					
1 week	72	49 (68)	23 (32)	<.001*	1.42, 1.09-1.83	.01
2 weeks	126	73 (58)	53 (42)	.14	1.21, 0.94-1.55	.08
3 weeks	100	48 (48)	52 (52)	Ref.	Ref.	Ref.
4 weeks	90	49 (54)	41 (46)	.37	1.13, 0.86-1.50	.41
5 weeks	42	27 (64)	15 (36)	.07	1.34, 0.99-1.81	.56
Accumulated radiation dose	n = 434	n = 247	n = 185			NA^b^
−14 2681	80 (18)	50 (62)	30 (38)	.16	1.17, 0.95-1.43	
14 2682-48 1195	280 (65)	150 (54)	130 (46)	Ref.	Ref.	
48 1196-	74 (17)	47 (64)	27 (36)	.13	1.19, 0.97-1,45	

n (number) and proportion (%) of patients are presented, n providing data is presented in case of missing data. Ref. = Reference category, relative risk 1.0; the category with the lowest proportion not practicing self-care. CI = 95% confidence interval.

a Other: students, unemployed, or housewife/husband.

b NA, Not applicable to be included in the multivariable analysis due to *P* was not <.10 in the univariable analysis.

c NA, Not applicable to be included in the multivariable analysis due to low n.

^d^NA, Not applicable due to that the variable was highly correlated to age groups.

* Statistically significant difference at 5% significance level.

** Fishers Exact Test.

### Documenting Patients’ Clinical Characteristics in Terms of Symptoms

The valid and reliable Swedish version^32^ of the Memorial Scale for Assessment of Symptoms (MSAS)^33^ asked the patients to grade the occurrence (“Yes” or “No”) of 32 different symptoms during the past week. Examples of the 32 symptoms are: lack of energy, worrying, feeling sad, nausea, vomiting, feeling drowsy, and problems with sleeping.

### Documenting Patients’ Self-care Practice

Based on the previously described face validity testing,^3^ the patients completed a clinimetric^31^ measure regarding self-care strategies: “Have you during the past week practiced any self-care strategies on your own to prevent or reduce symptoms?” (“No” or “Yes”). To capture the patients’ own perspective, a follow-up question posed: “If you practiced self-care, what kind of self-care was it and for what symptoms?” (patients answered in their own words).^15^

### Documenting Patient’s Functional Capacity and QoL

Regarding functional capacity, the patients answered the single-item question: “How many of your normal daily activities have you been able to perform during the past week?” (7-grade ordinal scale ranging from “All activities” to “None of my daily activities”). They also detailed their capacity to do household tasks, purchase food or other necessities, visit friends or relatives, and to walk on foot or get around. Detailed information on these measures has previously been presented.^3^ Regarding QoL-related characteristics, the patients reported their self-perceived health status using the Swedish version of the valid and reliable^34^ EuroQol-5 Dimension (EQ–5D), covering mobility, self-care, usual activities, pain/discomfort, and anxiety/depression. This was rated as 1; no problems, 2; some problems, 3; extreme problems. They also rated health status on the vertical Visual Analog Scale, EQ-VAS, from 0 (“worse possible health”) to 100 (“best imaginable health”).^35^ Further, the patients completed the valid and reliable Swedish version^36^ of the Functional Assessment of Cancer Therapy-General (FACT-G),^37^ which is widely used for measuring health-related QoL in cancer patients. They also graded their overall QoL on a numerical rating scale from 0 (“very poor QoL”) to 7 (“best possible QoL”), often used in patients with cancer to obtain a short clinimetric^31^ measure of overall QoL.^38^

### Data Analysis

When describing the patients, we presented descriptive statistics: number (n), percentages (%), median (md) with Inter Quartile Range (IQR) for ordinal variables, and mean value with standard deviations (SD) for continuous normally distributed variables. We summed the number of MSAS assessed symptoms that each patient had reported and categorized the summed number of symptoms into 0 to 7 symptoms, and 8 or more symptoms, based on the median value 8 symptoms (IQR 5-15). Numbers and percentages of patients practicing or not practicing self-care were summed and presented. To present the patients’ own perspective using categories, the different self-care strategies, described using the patients’ own words, were categorized using quantitative content analysis.^39^ Similar self-care descriptions were categorized into groups representing different self-care strategies. For instance, the descriptions “rested more often” and “take a break more frequently” were both categorized as “Increased recovery.” Similarly, “distract myself” and “watch funny movies” were both categorized as “distraction.” This was an inductive analysis used, to capture the patients’ own perspective. Accordingly, traditional integrative cancer therapy categories, for example, mind-body therapies, natural products, lifestyle changes^16^ were not used. The various indications for self-care were categorized in the same way, grouping similar indications to form categories.^40^ For example, the indications “to feel better” and “for maintaining my wellbeing” were both categorized as “general wellbeing.” As a first step, the second author made suggestions regarding the content analysis^40^ categories. In a second step, the first and last author reviewed the content analysis^40^ in relation to the content of the patients’ written descriptions. A few revisions were made; the categories were discussed until consensus was reached.

The proportion of patients practicing or not practicing self-care was presented for the total study group and for subgroups of patients with different sociodemographic and clinical characteristics. Chi-square-tests (Fishers’s exact test if n was <5) were used to compare these subgroups, presented as relative risks (RR) of not practicing self-care, with 95% confidence intervals (CI). The reference category was defined as the category with the lowest proportion not practicing self-care. We selected possible explanatory variables (all variables seen in Table 2 resulting in a *P*-value of <.10, according to the univariable analysis) that might explain the variation in proportions not practicing self-care, using a multivariable generalized logistic model. A response analysis was also conducted to ensure that any loss in the multivariable analysis did not affect its results.

**Table 2.** Self-care Strategies and Indications for Practicing Self-care, as Described by the Patients.

Described self-care strategies	Numbers of patients practicing each self-care strategy
Physical activity	113
Increased recovery	69
Healthy eating	66
Skincare	20
Distraction	24
Self-medication	12
Socializing	9
Oral care	4
Thinking positively and lowering demands	4
Toilet habits	3
Reducing intake of alcohol/tobacco	3
Routines	2
Massage	1
Professional guidance	1
14 strategies	332
Described indications for practicing self-care	Numbers of patients describing each indication
Fatigue	68
General wellbeing	29
Psychological symptoms	22
Nausea and vomiting	21
Improve physical condition	20
Urinary and bowel health problems	18
Skin problems	12
Pain	11
Oral health problems	5
Difficulties swallowing	2
Edema and swelling	2
Shortness of breath	1
Vertigo	1
13 indications	212

The table presents the categorization of self-care strategies and indications for practicing self-care, according to the patients’ own descriptions, together with the number of study participants who reported using the self-care strategy and for what indication.

Subsequently, we compared patients who practiced self-care and those who did not regarding functional capacity, and regarding QoL-related variables (health status graded on EQ-5D and EQ-VAS, and health-related QoL graded on FACT-G, sub-domains and total score, and overall QoL), using the Mann Whitney *U*-test. The significance level was set at *P* < .05. The data were analyzed using the Statistical Package for the Social Sciences (SPSS) software version 25 (IBM Corp, Armonk NY, USA).

## Results

**Figure 1.**
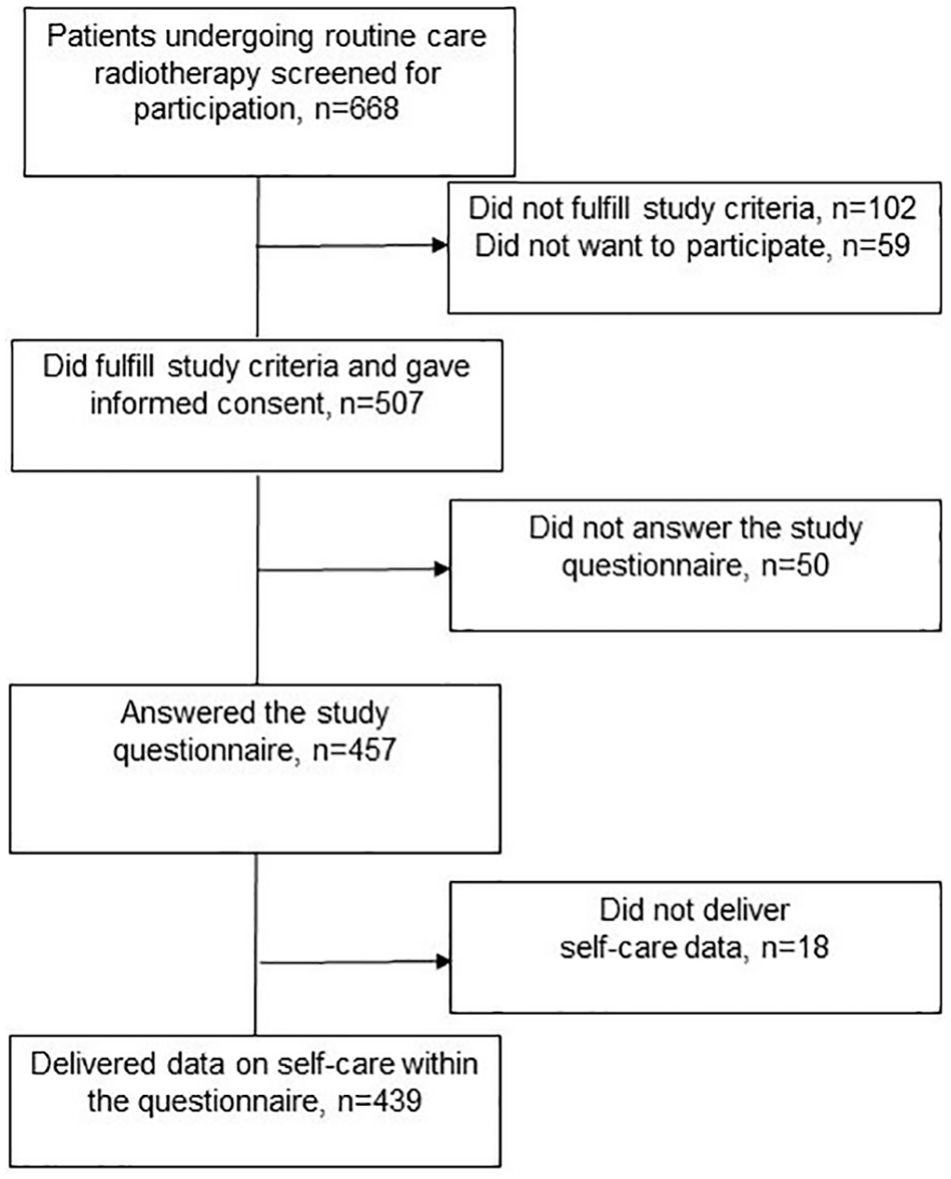
Flowchart of the number of patients screened, included, and providing data during radiotherapy.

The radiotherapy nurses initially included 507 patients, 457 of whom completed the study questionnaire. Of these 457 patients, 18 did not provide any self-care data. Accordingly, results are presented for 439 patients (Figure 1). The patients’ mean age was 66 ± SD 12 years old (range 19-91 years). On the study day, the patients had received an accumulated radiotherapy dose of mean value 33 ± SD 17 Gy. The radiotherapy was mostly combined with at least one other cancer therapy; n = 265 (58%) had undergone tumor surgery preceding the radiotherapy, and 157 (36%) had received concomitant chemotherapy. The patients were mostly treated for breast or prostate cancer. Table 1 presents the demographics and clinical characteristics of the patients.

### Self-care Practice in Patients With Different Sociodemographic and Clinical Characteristics

Of the 439 patients, 189 (43%) practiced at least one self-care strategy while 250 (57%) did not. Table 1 presents the differences between practitioners and non-practitioners of self-care. According to the multivariable analysis, the following characteristics were statistically significantly associated with lack of self-care practice: being age 69 or older, married, with an education-level lower than university, having undergone a combination of internal and external radiotherapy and having experienced fewer than 8 symptoms (Table 1).

### Self-care Practices

**Figure 2.**
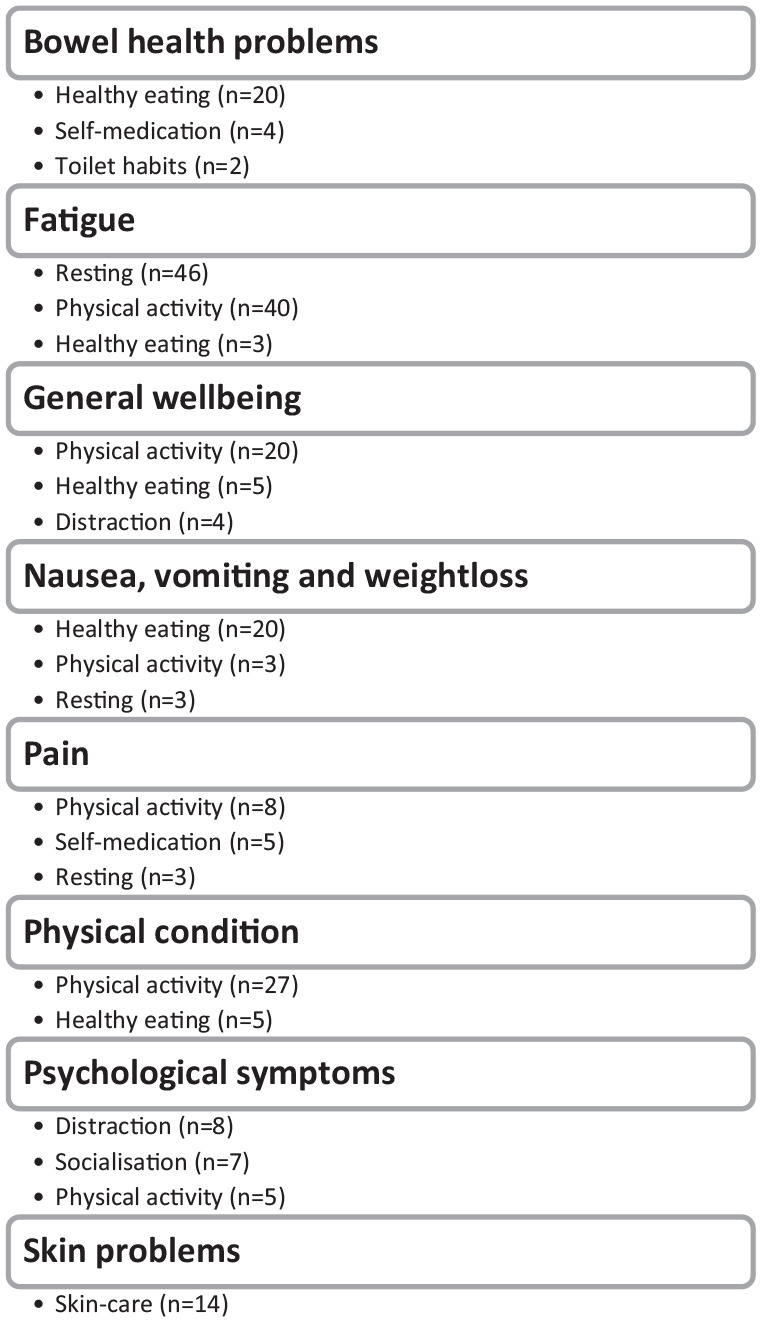
The most prevalent practiced self-care strategies, for a variety of indications.

**Figure 3.**
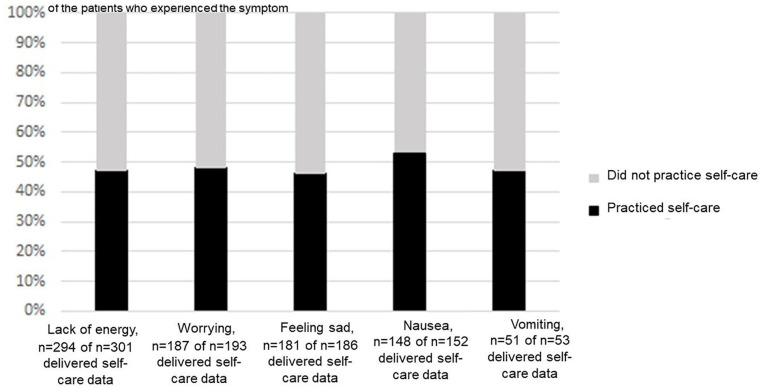
Self-care practice in patients experiencing the 5 most prevalent symptoms, assessed on the Memorial Scale for Assessment of Symptoms (MSAS).

In total, the 189 practitioners of self-care described 332 self-care practices, resulting in 14 different categories of self-care strategies. Each self-care practitioner described a median value of 1 (25th-75th percentile 1-2) self-care practice. The 5 most common indications for practicing self-care were fatigue (n = 68 patients reported practicing self-care), general wellbeing (n = 29), psychological symptoms (n = 22), nausea and vomiting (n = 21), and improving physical condition (n = 20). The 5 most practiced self-care strategies were physical activity (n = 113), improved recovery (n = 69), healthy eating (n = 66), distraction (n = 24), and skincare (n = 20) (Table 2 and Figure 2). The proportions of patients practicing self-care did not vary across patients experiencing the 5 most prevalent symptoms in this study group (Figure 3).

### Functional Capacity in Patients Practicing Compared to Patients Not Practicing Self-care

Of the patients, n = 432 provided data on general daily activities. A total of 136 (73%) of the patients who practiced self-care stated that they had been able to perform all or most of their daily activities during the past week. For those who did not practice self-care, 184 (75%) patients had been able to perform all or most of their daily activities during the past week. This difference was not statistically significant (*z* = −0.001, *P* = .999). There were no statistically significant differences between those who practiced self-care and those who did not regarding the ability to perform household tasks such as cooking, washing dishes or cleaning (*z* = 1.204, *P* = .23), the ability to purchase food or other necessities (*z* = −1.068, *P* = .29), or to walk on foot or get around (*z* = −0.865, *P* = .387). Those who did not practice self-care reported a greater social functional capacity to visit friends and family compared to those who did practice self-care (*z* = 2.256, *P* = .02).

### QoL in Patients Practicing Compared to Patients Not Practicing Self-care

Practitioners of self-care reported a worse EQ-5D score, with more problems with pain/discomfort and anxiety/depression compared to those who did not practice self-care. Self-care practitioners reported significantly poorer overall QoL and health-related QoL on FACT-G compared to non-practitioners, mainly related to lower levels of physical QoL (Table 3).

**Table 3.** Self-perceived health status and QoL in patients practicing or not practicing self-care.

	Group total	Practiced self-care	Did not practice self-care	Mean difference	*Z*-value^a^	*P*-value
EuroQoL, mean (±SD)						
EQ-VAS	68.54 (21.07)	66.85 (20.40)	69.80 (21.51)	2.96	−1.73	.08
Mobility dimension	1.16 (0.37)	1.14 (0.34)	1.18 (0.40)	0.46	−1.19	.24
Self-care dimension	1.01 (0.13)	1.01 (0.10)	1.01 (0.14)	0	−0.28	.78
Usual activities dimension	1.25 (0.55)	1.27 (0.54)	1.23 (0.53)	−0.04	−0.87	.38
Pain/discomfort dimension	1.62 (0.57)	1.72 (0.56)	1.55 (0.57)	−0.17	−3.10	<.001*
Anxiety/depression dimension	1.46 (0.56)	1.56 (0.60)	1.39 (0.53)	−0.18	−3.12	<.001*
EQ-5D index score	0.77 (0.23)	0.74 (0.23)	0.80 (0.22)	−0.06	−3.1	<.001*
FACT-G, mean (±SD)						
Physical QoL	22.01 (5.3)	21.18 (5.26)	22.64 (5.24)	1.46	−3.4	<.001*
Social QoL	23.24 (4.79)	22.98 (4.48)	23.43 (5.00)	0.47	−1.67	.1
Emotional QoL	19.16 (4.18)	18.80 (4.45)	19.43 (3.96)	0.64	−1.2	.23
Functional QoL	17.27 (5.58)	17.07 (5.21)	17.43 (5.84)	0.36	−1.08	.28
Total score QoL	81.83 (15.04)	80.23 (15.03)	83.02 (14.97)	2.8	−2.03	.04*
Overall single-item measured QoL, median (25-75 percentile)						
Overall QoL	6 (5-7)	6 (4-7)	6 (5-7)	-	−1.8	.07

Abbreviations: EQ-VAS, EuroQoL Visual Analog Scale; EQ-5D, EuroQoL 5 Dimensions; FACT-G, Functional Assessment of Cancer Therapy-General; QoL, Quality of Life.

Higher scores indicate better health and QoL.

a Mann-Whitney *U*-test.

* Statistically significant difference at 5% significance level.

## Discussion

The present study showed that slightly less than half of the patients practiced self-care during a regular week of radiotherapy, the most common self-care strategies were physical activity, increased recovery, healthy eating, distraction, and skincare. The indications for self-care practice were mostly fatigue, general wellbeing, psychological symptoms, nausea and vomiting, and improved physical condition. Patients who experienced more symptoms and poorer QoL were more likely to practice self-care, while functional capacity did not differ between self-care practitioners and non-practitioners. Elderly patients and patients with a lower level of education were less likely than other patients to practice self-care.

The proportion of patients practicing self-care in the present study is in accordance with figures from other studies,^39^ which estimated that 40% of all cancer patients used different self-care strategies, especially as a complement to medications for radiotherapy-induced side effects. In other studies,^41-43^ the proportion of self-care practitioners was higher, up to 95%, or lower, 25%.^15^ One factor that makes it difficult to estimate the proportion of self-care practitioners is that not everyone is aware the activities they are performing to increase wellbeing are in fact self-care activities. Despite the great differences in use of self-care, however, there seems to be a trend: The practice of integrative cancer therapies has increased over time.^20,44^

The patients in our study mostly practiced the self-care strategies physical activity, increased recovery, healthy eating, and distraction. Based on strong scientific evidence showing that the self-care strategy physical activity reduces symptoms and improves health,^45^ it was expected that physical activity would be one of the most commonly practiced strategies. Specially to reduce fatigue, many patients practiced physical activity, while we had expected the numbers of patients practicing this self-care activity for pain and psychological symptoms to be higher in light of the solid evidence.^45^ Besides physical activity, studies have shown that healthy eating and increased recovery are other strategies previously applied during radiotherapy. Of the 200 studied patients, 25% practiced self-care for radiotherapy-induced nausea, mostly by changing eating or drinking habits, increasing frequency of rest and recovery, or being physically active.^15^ What kind of healthy eating habits efficiently reduce nausea and bowel health problems is still to be researched; there are still few high-quality studies conducted in this area.^25,46,47^ There is low-certainty evidence that dietary counseling reduces radiotherapy-induced bowel health problems.^25^ However, in general, several studies have shown promising effects of self-care.^20,23-27^ One review^27^ found that 75% of the included trials revealed a positive effect of the self-care activities physical activity, cognitive distraction, and increased recovery, that is, relaxation. Overall, there appears to be at least one effective self-care strategy for each of the symptoms^20^ experienced by the currently studied patients. It therefore seemed valuable for several reasons to study which of those strategies were practiced, as well as what symptoms the strategies targeted. Our findings generated knowledge indicating that scientific evidence on effective self-care strategies for different symptoms has been partially integrated^16^ into and implemented^30^ in routine care. Further, because integrative cancer care is a patient-centered, evidence-informed field of cancer care,^16^ the present findings also offer patient suggestions on various self-care strategies that require further scientific evaluation concerning their safety and efficacy. As mentioned, few high-quality studies have evaluated the effects of healthy eating on symptom experience,^25,46,47^ although this kind of self-care strategy is commonly practiced and suggested by cancer care practitioners.^26^ The present study showed that slightly more than half of the patients did not practice any self-care at all even though effective self-care strategies exist.^20,48^ Therefore, it would seem important to have a dialog in which the patient and healthcare practitioner share relevant information, discuss the risks or burden versus the benefits of different self-care strategies, express preferences, consider alternatives and agree on treatment in the shared decision-making procedure.^48^A previous study found that patients who were offered a dialog with healthcare practitioners through an e-health device during their chemotherapy or radiotherapy period (n = 149) showed great engagement in their self-care management.^49^

We found that the most commonly observed indications for self-care practice were fatigue, general wellbeing, psychological symptoms, nausea and vomiting, and improvement in physical condition. These indications were, as expected, in line with the most commonly experienced symptoms in patients undergoing radiotherapy.^4,6,10,11^The proportions of patients practicing self-care did not vary across patients experiencing the most prevalent symptoms in this study group, proposing that self-care was used for the experienced clusters of symptoms. Several factors have been proposed to underlie the use of self-care. Self-care is perceived to have a positive effect on perceived feeling of control over symptoms,^20,29,50^ alongside the poor or lacking effect of conventional medical treatment.^7,50^ Other reasons may be that symptom intensity is not great enough to require professional care,^50^ that healthcare practitioners under-estimate the symptoms,^18^ and that self-care is easily accessible.^51^ Still other reasons are lack of healthcare remedies in combination with a strong desire for relief and remedies, as well as the emergence of other care and support needs.^28,29^ Patients who experienced more symptoms, as well as poorer health and QoL, were more likely than other patients to practice self-care, indicating that patients practiced self-care to relieve symptoms, not to prophylactically prevent symptoms. It was expected that self-care practitioners would be the patients who experienced many symptoms and worsened health and QoL, as symptoms often co-occur and together interfere with several different aspects of health and QoL.^9^ Elderly patients and patients with a lower level of education were less likely to practice self-care compared with other patients, even after adjusting for the number of symptoms experienced. Lower level of education also predicted a decrease in engagement in self-care management in a previous study conducted during chemotherapy or radiotherapy.^49^ Our age and education-related findings may be discussed in light of the fact that both factors tend to be of great relevance to the level of health competence.^52^ The fact that it was the older age group that practiced less self-care may be an expression of a generational issue, in that older patients have experienced a different hierarchy in healthcare during their lifetime and have relied on care that does not entail their own participation. Our descriptive results, only statistically significant according to the univariable analyses, indicated that patients used self-care to a lesser extent at the beginning and end of their radiotherapy period. This may be important knowledge for healthcare practitioners; patients may need the practitioner to pay extra attention to their self-care practice during these treatment periods.

The present findings provide a consistent message: Patients undergoing radiotherapy experience a high frequency of symptoms and almost half showed engagement in managing symptoms using self-care. Given the consistency of these results, perhaps it is time to focus research on how integrative cancer care can best benefit patients during this period of discomfort. The next step, and perhaps the most important one, will be to reach out and communicate this with subgroups of patients who need more support that enables them to engage in self-care. Some researchers have suggested that a case management model may be useful in increasing engagement in and the efficacy of self-care among patients undergoing radiotherapy.^53^ Others suggested that e-health devices are preferable.^23,26,49^ Several studies have shown that patients have difficulties initiating communication about self-care strategies with healthcare practitioners and that this is a source of dissatisfaction.^51^ Patients do not feel comfortable talking about their use of self-care with healthcare practitioners, who in turn do not ask patients about self-care.^54^ Relatives, family members and friends have been identified as an important source of information regarding self-care knowledge.^50^ To get patients and healthcare practitioners involved, questions about routine use of self-care should be included in the shared decision-making procedure.^48^ Health practitioners may need more education in integrative oncology to be able to support the patients.^16^ Finally, this is a matter of long-term trust in the healthcare system, but above all of reducing suffering and increasing QoL, in line with the goals of integrative cancer care.^16^

Striving to secure the validity of the study and thus avoid bias in its different steps, we adhered to the hierarchical step model designed for this purpose.^55^ Regarding minimization of bias related to the first step, person-time, the relationship between the independent variables educational status and age and the dependent variable practice of self-care was still valid after adding the summed number of symptoms to the analysis. This was important as it is reasonable to assume that having more symptoms would prompt patients to practice self-care. The cross-sectional design of the data collection enabled us to collect data during a limited period of calendar time but with varying lengths of follow-up time. Accordingly, the patients presented heterogenicity regarding accumulated dose and duration of radiotherapy at the time of data collection. A longitudinal approach would have allowed us to observed indications of the direction of the relation between self-care and the various symptoms experienced by patients. Patients who experienced more symptoms, as well as poorer health and QoL, were more likely than other patients to practice self-care. Interpreting this outcome seemed easy; Patients practiced self-care to relieve symptoms and improve health. If the opposite had occurred, that is, if self-care practitioners had experienced fewer symptoms, as well as better perceived health and QoL, we would (due to the cross-sectional design) not have been able to determine whether patients with fewer symptoms and better health had a better capacity to practice self-care, or whether the self-care *per se* seemed to affect symptom experience. However, our study was not intended to evaluate any effects of self-care practice. Instead, we merely observed the practice in this routine radiotherapy setting, our aim being to identify subgroups in need of more support. The cross-sectional design enabled us to depict the trajectory of self-care without the measurement-induced bias that may occur when a certain phenomenon is measured repeatedly in the same patient. Regarding the second step, misrepresentation,^55^ 2 strengths of our study are that the patient sample size was large and that the response rate for the self-care data was high, 87%. We have no information about the patients who did not respond to the questionnaire at all. Regarding the 18 of the 457 study patients who did not specifically provide self-care data, their sex and age distribution (data not presented in results, just for discussion) was similar to that of the responders. They may, or may not, have been non-practitioners of self-care to a higher extent than the responders were. The third step of the hierarchical step model^55^ covers bias induced by misclassification due to incorrect data. An important part of our study was thus the validity and reliability of the clinimetric^3,31,38^ and psychometric^33-37^ measures in the study questionnaire. We found it important to adopt previously used data collection methods regarding self-care,^15^ symptoms,^32,33^ daily activities,^3^ health,^34,35^ and QoL.^36,37^ By employing validity, reliability^3^ and pilot testing, as well as through previous use^15^ of the measure, we ensured that the self-care measure was well understood and not too difficult to answer. Without this pre-testing, we could have interpreted the higher proportion of non-practitioners of self-care as being a result of measurement-induced bias. We might have thought that patients in the older age group did not label their activities as self-care, thus practicing self-care without thinking of it as such. To reduce potential bias induced by information in the questionnaire and to capture each individual patient’s perspective, the questionnaire provided no list of self-care strategies. The patients described their self-care strategies using their own words. The patients received the study questionnaire from radiotherapy nurses who clearly declared that the answers would not be read by them; patients completed the questionnaire in private and sent it to the evaluator. It can reasonably be assumed that these data collection routines lowered the potential risk of therapist-induced bias. Regarding the fourth and last step of the hierarchical step model,^55^ the statistical analyses, we did not include the variables daily activities, health, and QoL in the analysis as factors that could possibly explain the variation in self-care, because we do not know the direction of these relationships. Besides numbers of symptoms, we only selected characteristics that could reasonably be assumed not to be consequences of self-care. No sample size calculation was made based on the self-care measure. The sample size calculation was based on the possibility to detect differences between the group of patients and matched referents from the Swedish general population, in a future long-term follow-up after radiotherapy. We estimate the risk of type-2 errors to be low as the observed differences, in absolute figures, between self-care practitioners and non-practitioners reached statistical significance. Integrative cancer care is a patient-informed field.^16^ Accordingly, the inductive categorization of self-care strategies represents the patients’ own perspective, described using their own words. Accordingly, traditional integrative cancer therapy categories, for example, mind-body therapies, natural products, lifestyle changes^15^ were not applied in a deductive way when categorizing of the self-care strategies. There appears to be at least one effective self-care strategy for each of the symptoms^20,23,26^ experienced by the studied patients and the proportions of patients practicing self-care did not vary across patients experiencing the 5 most prevalent symptoms in this study group. Accordingly, we found it reasonable to categorize the patients into the 2 groups, self-care practioners and non-practioners. This choice regarding the categorizing made it possible to contribute knowledge regarding subgroups that did not practice any self-care at all, and that thus require more support. The mean age of our patients was 66 years and most patients received radiotherapy for breast or prostate cancer, which is in line with the most prevalent cancer diagnoses. One limitation is thus that this naturally lowers the generalizability of the present findings to younger patients and patients with rarer types of cancer.

## Conclusion and Implications for Practice

The present study found that slightly less than half of patients practiced self-care during an ordinary week of radiotherapy, and that the most frequently used self-care strategies were physical activity, increased recovery, healthy eating, distraction, and skincare. The primary indications for self-care practice were fatigue, general wellbeing, psychological symptoms, nausea, and vomiting, and improving physical condition. Patients experiencing more symptoms as well as poorer health and QoL were more likely than other patients to practice self-care. Because older patients, and patients of all ages with a lower level of education were less likely than other patients to practice self-care, healthcare practitioners in integrative cancer care should consider paying particular attention to supporting these subgroups of patients in the use of evidence-based self-care strategies.
